# Feasibility of a dried blood spot strategy for serological screening and surveillance to monitor elimination of Human African Trypanosomiasis in the Democratic Republic of the Congo

**DOI:** 10.1371/journal.pntd.0009407

**Published:** 2021-06-11

**Authors:** Raquel Inocencio da Luz, Delphin Mavinga Phanzu, Oscar N’lemvo Kiabanzawoko, Eric Miaka, Paul Verlé, Anja De Weggheleire, Philippe Büscher, Epco Hasker, Marleen Boelaert

**Affiliations:** 1 Institute of Tropical Medicine, Antwerp, Belgium; 2 Centre de Recherche en Santé de Kimpese, Kimpese, Democratic Republic of the Congo; 3 National Sleeping Sickness Control Program, Kinshasa, Democratic Republic of the Congo; Universiteit Antwerpen, BELGIUM

## Abstract

In recent years, the number of reported Human African Trypanosomiasis (HAT) cases caused by *Trypanosoma brucei (T*.*b*.*) gambiense* has been markedly declining, and the goal of ‘elimination as a public health problem’ is within reach. For the next stage, i.e. interruption of HAT transmission by 2030, intensive screening and surveillance will need to be maintained, but with tools and strategies more efficiently tailored to the very low prevalence. We assessed the sequential use of ELISA and Immune Trypanolysis (ITL) on dried blood spot (DBS) samples as an alternative to the traditional HAT field testing and confirmation approach. A cross-sectional study was conducted in HAT endemic and previously endemic zones in Kongo Central province, and a non-endemic zone in Haut Katanga province in the Democratic Republic of the Congo (DRC). Door-to-door visits were performed to collect dried blood spot (DBS) samples on filter paper. ELISA/*T*.*b*. *gambiense* was conducted followed by ITL for those testing positive by ELISA and in a subset of ELISA negatives. In total, 11,642 participants were enrolled. Of these, 11,535 DBS were collected and stored in appropriate condition for ELISA testing. Ninety-seven DBS samples tested positive on ELISA. In the endemic zone, ELISA positivity was 1.34% (95%CI: 1.04–1.64). In the previously endemic zone and non-endemic zone, ELISA positivity was 0.34% (95% CI: 0.13–0.55) and 0.37% (95% CI: 0.15–0.60) respectively. Among the ELISA positives, only two samples had a positive ITL result, both from the endemic zone. One of those was from a former HAT patient treated in 2008 and the other from an individual who unfortunately had deceased prior to the follow-up visit. Our study showed that a surveillance strategy, based on DBS samples and centralized testing with retracing of patients if needed, is feasible in DRC. ELISA seems well suited as initial test with a similar positivity rate as traditional screening tests, but ITL remains complex. Alternatives for the latter, also analyzable on DBS, should be further explored.

## Author summary

Human African Trypanosomiasis (HAT), also known as sleeping sickness is a parasitic disease, transmitted by tsetse flies, that is usually fatal if untreated. The number of cases have been rapidly declining over the past years indicating that elimination of the disease as a public health problem is within reach. To achieve the next stage, i.e. interruption of HAT transmission by 2030, intensive screening and surveillance will need to be maintained, but with tools and strategies more efficiently tailored to the very low prevalence. In contrast to the traditional approach of sending laboratory expertise to the field, we assessed an alternative approach based on the collection of dried blood samples on filter paper that were tested in a regional laboratory. Samples were taken in endemic, previously endemic and non-endemic villages and tested by ELISA and Immune Trypanolysis. The ELISA positivity rates were similar to those of other screening techniques currently used and Immune Trypanolysis was highly specific. Hence for surveillance in HAT endemic areas, collecting dried blood samples followed by centralized testing could become an alternative to the current strategy of active screening by mobile teams with on the spot confirmation. It has also potential for post-elimination surveillance to monitor resurgence and for exploratory surveillance in historic foci. Though highly specific, Immune Trypanolysis remains too complex for use in intermediate level laboratories, to further expand this strategy an alternative second step test is required.

## Introduction

In Western and Central Africa, Human African Trypanosomiasis (HAT), also known as sleeping sickness, is caused by *Trypanosoma brucei (T*.*b*.*) gambiense* and transmitted by tsetse flies. The disease has two stages: the first is characterized by trypanosomes that multiply in the peripheral organs and tissues, the second evolves when the central nervous system is invaded [1]. Clinical signs and symptoms are unspecific during the first stage and can last for several years. Neuropsychiatric symptoms such as sleep and behavioral disturbances that lead to coma and death, are characteristic of the second stage [2]. Without adequate treatment, the disease is fatal in most cases.

The WHO has developed a road map with the goal to eliminate HAT as a public health problem by 2020 [3]. The main global indicator, i.e. <2,000 cases reported at continental level, has been amply met. The number of reported cases in Africa has declined from 9,680 in 2009 to fewer than 1,000 cases by 2019 (n = 992) including both *T*.*b*. *gambiense* and *T*.*b*. *rhodesiense* [4]. Also the second target set at a 90% reduction from 2004 baseline levels of the total areas at high risk (*i*.*e*. reporting >1 case/10,000 people/year) is considered within reach [3]. Although some countries have not yet met the second elimination goal, overall progress is remarkable, and this brings the next goal which focuses on interruption of HAT transmission in humans by 2030 in view.

Historically, the Democratic Republic of the Congo (DRC) bears the heaviest burden of the disease, reporting between 65 and 85% of all HAT cases [3]. For decades, the DRC control strategy has been based on active screening of the inhabitants of villages at risk using specialized mobile teams. These teams perform on the spot screening and diagnostic confirmation of entire village populations. However, with rapidly declining endemicity, this costly and labor-intensive strategy becomes harder to justify. Individuals can also present themselves at fixed health facilities, the so-called passive detection, but considering the poor performance and utilization of the health system in DRC, this strategy on its own is not a reliable alternative for further progress. An important drawback of passive detection is the risk to diagnose HAT cases at a later stage of the disease, implying that the time they act as reservoirs for tsetse flies is prolonged [5].

In both active and passive detection, diagnosis is a two-step process based on serological screening for gambiense HAT (gHAT) antibodies, followed by a confirmation test to visualize the parasite in blood, lymph node aspirate or cerebrospinal fluid [6]. Different serological tests exist, using Variant Surface Glycoproteins (VSG) such as LiTat 1.3 and/or LiTat 1.5, which are either purified from the blood of rats infected with trypanosomes or (recently) produced as recombinant antigens.

Currently, the national HAT control program of DRC uses the Card Agglutination Test for Trypanosomiasis (CATT) on whole blood and Rapid Diagnostic Tests (RDT) for the serological screening. CATT is well fit for mass screening in villages: it allows a high throughput, is cheap, and delivers quick results so that confirmatory testing for seropositive individuals can be done on the spot. However, it requires some basic equipment which the person performing the test must know how to operate, a cold chain, 12 Volt electricity supply and related logistics. It is produced in vials of 50 test units that cannot be kept for more than one day once opened. RDTs are more suited for individual screening in fixed health facilities as they are individually packed, thermostable, instrument-free and easy to perform even by non-laboratory technicians. However, drawbacks experienced over the last five years were a less stable supply chain for some of the RDTs, and a higher cost than CATT in absence of donor subsidies [7]. Recently, a new RDT was developed using recombinant antigens, however, it is not yet available in DRC [8].

The specificity of both CATT and RDT is very high but not 100%, as a result of the declining prevalence the positive predictive values of both tests are getting increasingly low [8,9]. For epidemiological follow-up and monitoring of elimination in a given area or population, it is thus critical to identify whether there are still true cases. In 2019, only 1.8% of CATT positive individuals was confirmed as true HAT case in the former Bandundu province, considered the most endemic in DRC (PNLTHA, annual report 2019). False positive results for RDTs have been reported to be even slightly more common than for CATT [8,9]. The confirmation tests in the current testing algorithm are thus essential but require availability of technical expertise and specialized supplies at point of care. It is questionable whether this expertise can be maintained in the elimination context.

The very low prevalence and consequent focus on the interruption of transmission also pose the challenge of sustainability. It becomes harder to find donors to fund the costly mobile teams, even if some improvements have been made in the recent past [10]. We should not forget that we have been in this situation before. In the 1950s, intense control measures also had reduced the incidence of the disease to very low levels, but when the control measures relaxed, a general resurgence of HAT was observed in Central Africa, reaching alarming levels in the 1990s [11]. Endemic foci of gHAT have proven to be persistent, characterized by a basic reproduction number (Ro) that remained around 1, resulting in high halving or doubling times (To) [11]. Consequently, a resurgence of a historical focus can occur several years after the last case was detected. Early detection of such resurgences is crucial to obtain sustainable elimination as a public health problem and to advance further towards interruption of transmission. However, it should be avoided that false positive results trigger unnecessary alarm and ensuing actions. New screening and surveillance tools and new control strategies, which take these challenges and opportunities into account have to be developed and validated. Using easily collectable and transportable samples such as dried blood spots (DBS) and centralizing testing to regional or central laboratory levels could be considered.

Past evaluations of several HAT diagnostics (e.g. micro-CATT, LATEX / *T*.*b*. *gambiense* and enzyme-linked immunosorbent assay (ELISA)/ *T*.*b*. *gambiense*) on DBS samples showed encouraging results[5]. We assessed prospectively whether the sequential use of two serological tests performed on DBS samples is feasible and whether its performances are satisfactory in the context of very low endemicity. The ELISA/*T*.*b*. *gambiense* was selected for screening, the immune trypanolysis assay (ITL) was used to confirm the results [12–15].

## Methods

### Ethics statement

This study received ethical clearance from the Ethics Committee of the University Hospital of Antwerp, Belgium (B300201733044), and the Ethics Committee of the Université Protestante au Congo (CEUPC0046).

We obtained written informed consent from all participants, parents or legal guardian provided written informed consent for minors, but for minors between 12–17 years an additional written assent was obtained. Community workers ensured that the participants were duly informed about the objectives, procedures, potential risks and benefits, and the concept of voluntary participation. For HAT cases that would have been detected during the study, we had foreseen to facilitate referral to the nearest treatment center of the national sleeping sickness control program.

### Study design and setting

We conducted a cross-sectional study with data and sample collection in the provinces of Kongo Central and Haut Katanga, DRC. The province of Kongo Central (previously called Bas-Congo) is divided into 31 health districts covering each a population of about 100,000. Each health district is subdivided into health areas (HA) that cover several villages with populations of about 5,000–6,000 in total. Historically, Kongo Central was highly endemic for gHAT, but as a result of several decades of HAT control the number of reported cases has rapidly declined [16]. Over the last five years, cases have only been reported in 18 of the 31 health districts. In 10 districts no new cases have been reported for over ten years (source: national HAT control program DRC). Health areas are classified as *endemic* (*i*.*e*. HAT cases reported within the last 5 years) or *previously endemic* (*i*.*e*. HAT cases reported >5 years ago) (source: national HAT control program, DRC).

For the study, six endemic health areas were selected in Kimpese health district and two in Nsona Mpangu health district (Table 1). The health area of Lovo in Kimpese health district was selected as previously endemic health area, where no cases have been reported since 2007. All these health areas were rural, except for the semi-urban district of Songololo. As non-endemic control, the Kafubu health district of the province of Haut Katanga was selected, where no HAT case has ever been reported. This was necessary, because in all health areas of the province of Kongo Central, HAT cases have been reported previously. The control sample collection was organized in the health area of Adra 41.

**Table 1:** Overview of the study sites and HAT endemicity level.

Province	Health District	Health area by HAT endemicity level (n reported cases*)
Kongo Central	Kimpese	*Endemic*: Malanga(4), Viaza(5), Kilueka(2), Kizulu(0), Nkuanza(4), Vila(7)
		*Previously endemic*: Lovo
	Nsona Mpangu	*Endemic*: Songololo(1), Nsona Mpangu(0)
Haut Katanga	Kafubu	*Non endemic*: Adra41

* Health areas are considered endemic when a case was reported in the previous five years, the number of reported cases shown here date from the year prior to the fieldwork.

In the selected health areas, villages were randomly included until around 3,000 participants were enrolled for each represented epidemiological focus (endemic, previously endemic, non-endemic). For each included village, the entire population of the village was invited to participate and door-to-door visits were performed (one passage/household). Participants of 82 villages were enrolled: 67 in Kongo Central and 15 in Haut Katanga.

### Sample and data collection

**Fig 1:**
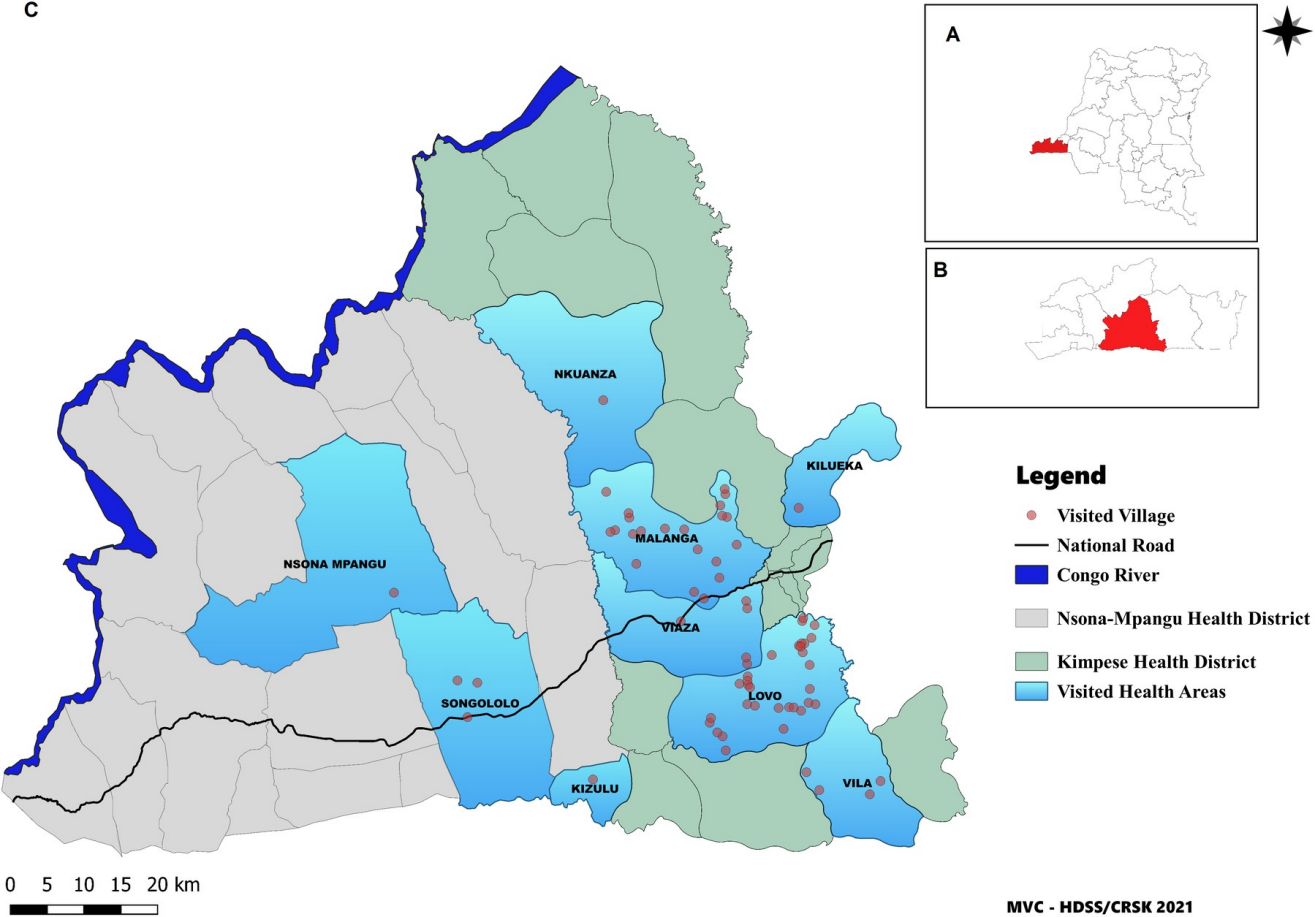
Map of the study area in Kongo Central Province: **A:** The Democratic Republic of the Congo with Kongo Central province highlighted. **B:** Kongo Central province, the study area highlighted. **C:** Map of Nsona Mpangu and Kimpese district showing all visited villages. Map created using QGIS 3.16 based on https://www.naturalearthdata.com/downloads/10m-cultural-vectors/ and https://www.diva-gis.org/gdata.

**Fig 2:**
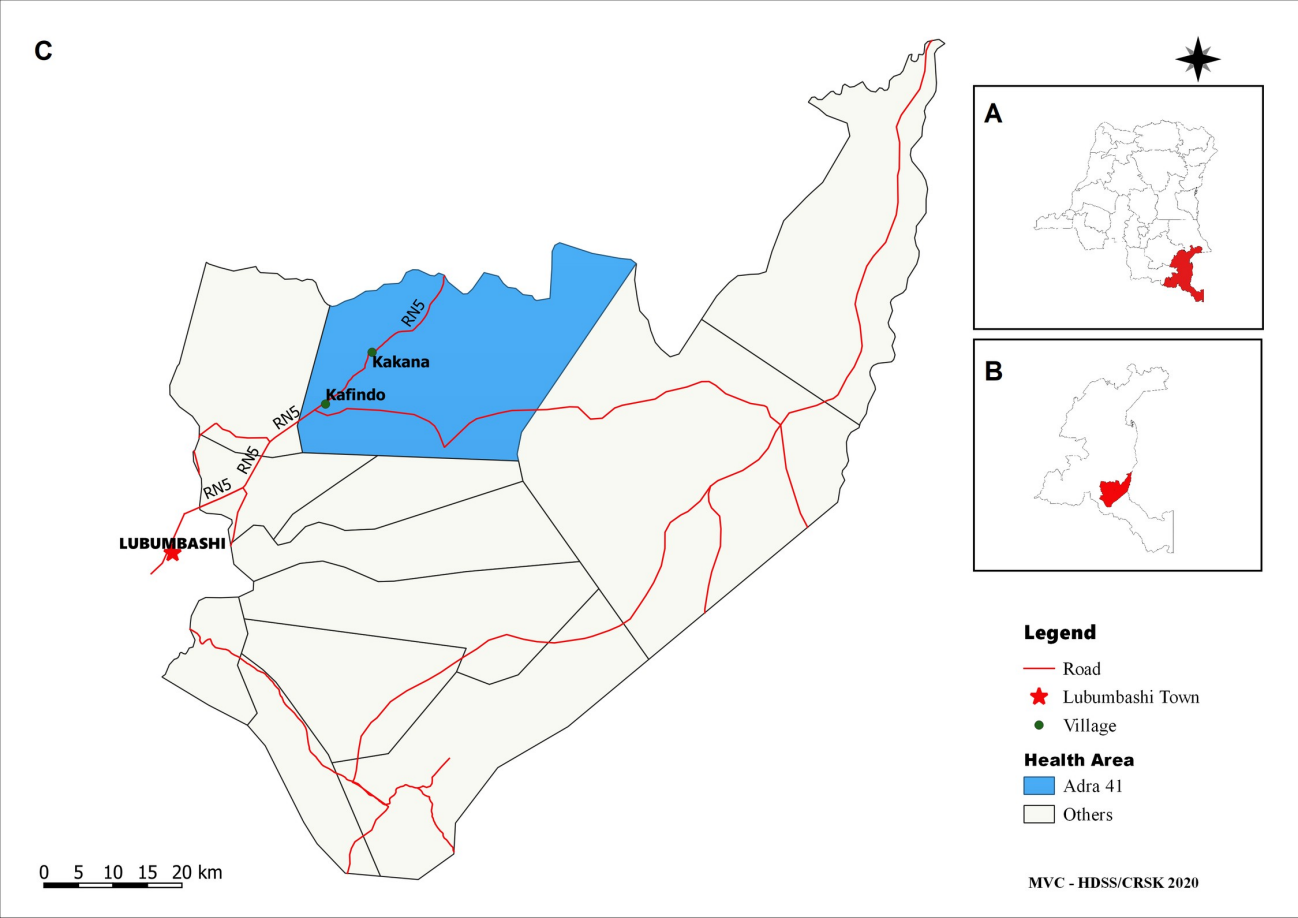
Map of the non-endemic are in Haut Katanga Provine: **A:** The DRC with Haut Katanga province highlighted. **B:** Health District of Kafubu is highlighted **C:** Health District of Kafubu, showing the health area Adra41. Map created using QGIS 3.16 based on https://www.naturalearthdata.com/downloads/10m-cultural-vectors/ and https://www.diva-gis.org/gdata

**Fig 3:**
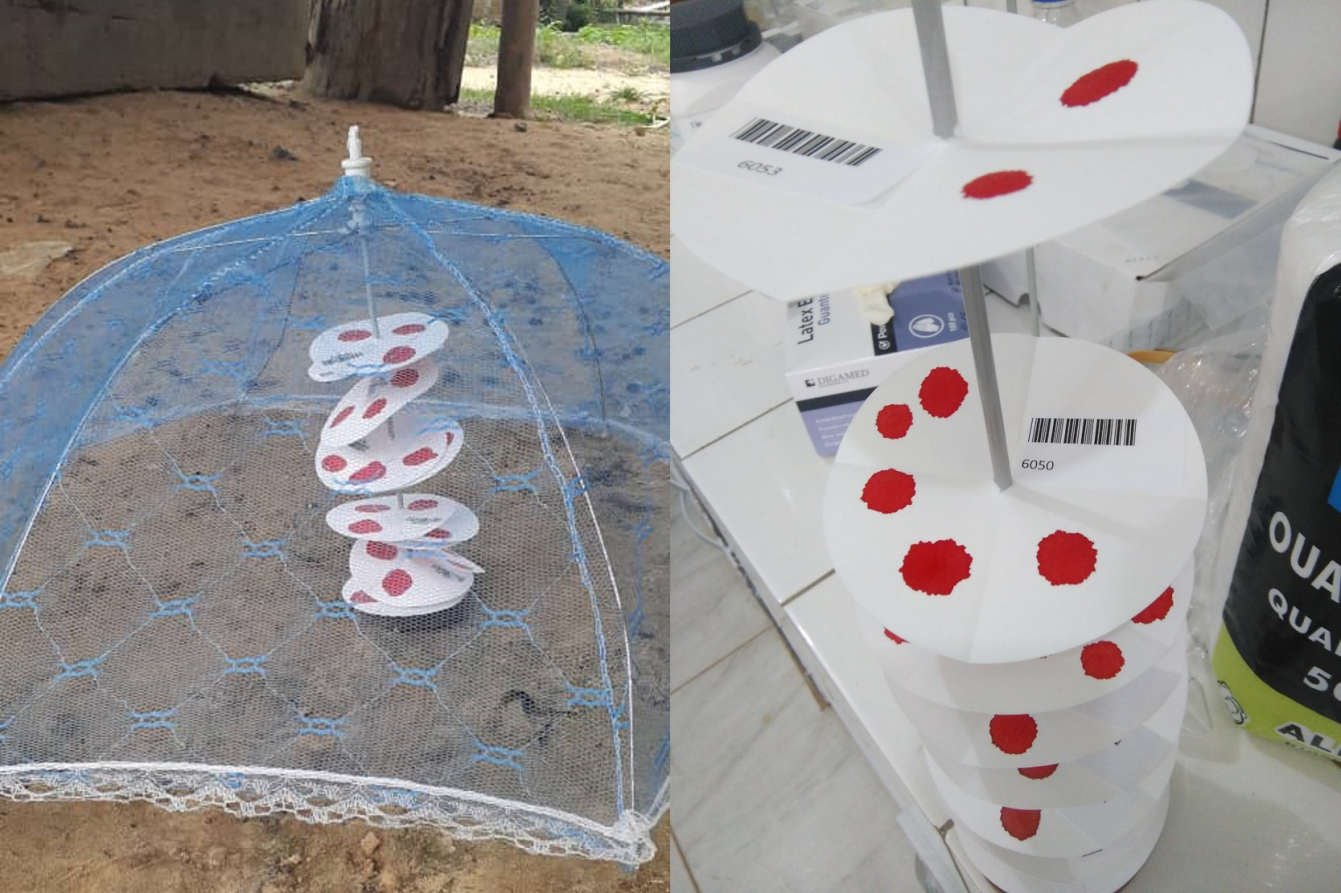
Drying of DBS samples on Filter Paper in the field, prior to transportation to the central laboratory.

DBS sample on filter paper and linked socio-demographic data collection was spread over a two year period between 2017 and 2019. In November 2017, samples were collected in the health areas of Malanga, Viaza and Lovo. In August 2019, the sampling collection was organized in the health areas of Kilueka, Nkuanza, Viaza and Vila (Kimpese health district) and the health areas Songololo, Kizulu and Nsona Mpangu (in Nsona Mpangu district) (Fig 1). The non-endemic health area of Adra 41 was visited in June 2018 (Fig 2). The duration of blood sampling activities was generally about two to three weeks per health area. Transport of the samples to the regional laboratory depended on the distance. Samples originating from the vicinity were transferred at the end of the day, while samples from remoter areas were stored on-site and transferred to the laboratory after completion of sampling in the health area. Field storage generally lasted no longer than three weeks. Field teams consisted of two individuals. One person was responsible for registering the participants (name, age, sex and place of residence) using an Android app developed in ODK collect and to assign a study code based on a pre-printed barcode sticker stuck on the DBS filter paper. As a back-up, participants were also registered on paper. The other team member was responsible for the blood sample collection. The blood sample was obtained from a finger prick. A maximum of 70μl of capillary blood was collected in two heparin coated capillary tubes and blotted directly or with the aid of a suction bulb on the Whatman 4 filter paper (two drops of about 35μl from each capillary tube). The drops were allowed to dry on a rack, composed of a pin mounted on a piece of wood/plastic, sheltered from the sun, flies and other insects (Fig 3). Once completely dry (dark brown color), 10 filter papers were put in one envelope and subsequently in a zip-lock bag containing 35g of silica gel (VWR) with color indicator. The bags were closed airtight, stored at ambient temperature and transported to the regional laboratory of the *Centre de Recherche en Santé de Kimpese* (CRSK) for analysis. Silica gel was replaced each time when the orange color of the indicator changed, indicating that no moisture could be absorbed anymore.

Individuals with a positive ITL result were revisited for a full HAT confirmation assessment on site, this follow-up visit could be 6 to 12 months after the initial sampling. The assessment consisted of a questionnaire to obtain information on HAT history, as well as work and migration habits of the individual. Next, the confirmation consisted, as per national program routine, of the microscopic examination of lymph node aspirate (in the case of typical lymph nodes) and/or the examination of a venous blood sample using the miniature Anion Exchange Centrifugation Technique (mAECT) on 500μl whole blood.

### Study laboratory procedures

We foresaw a sequential two-step testing algorithm, starting for all samples with the ELISA/*T*.*b gambiense*, followed by ITL for those testing positive to ELISA. Shipment and testing by ITL was also planned for a random sample of 10% of ELISA negative samples.

The ELISA/*T*.*b*. *gambiense* was performed in the CRSK laboratory in Kimpese as described elsewhere [12,17] with small modifications. One disc of 6mm diameter was punched out of the dried blood spot and left overnight in 720 μl PBS-Blotto (0.01 M phosphate, pH 7.4, 0.2 M NaCl, 0.05% w/v NaN3, 1% w/v skimmed milk powder; Fluka, Buchs, Switzerland), containing 0.05% v/v Tween 20 (Merck, Schuchardt, Germany). Microplates (Maxisorp, Nunc, Roskilde, Denmark) were coated overnight with 150 μL/well of a mixture of purified variable surface glycoproteins of *T*.*b*. *gambiense* LiTat 1.3, LiTat 1.5, at a concentration of 1 μg/mL [17]. Antigen free control wells were left empty. Plates were blocked for 1 hour at ambient temperature, with 350 μL/well of PBS-Blotto (0.01 M phosphate, pH 7.4, 0.2 M NaCl, 0.05% w/v NaN3, 1% w/v skimmed milk powder; Fluka). For testing, antigen containing and antigen-free wells were filled with 150 μL of eluate (in duplicate). A strongly positive control serum, diluted 1:150 in PBS-Tween, was tested in quadruple in each plate. The plate was incubated for 1 hour and washed 3 times with 350 μL/well of PBS-Tween (0.01 M phosphate, 0.14 M NaCl, 0.05% v/v Tween 20, pH 7.4). Goat anti-human IgG (H+L) peroxidase (Jackson Immuno Research, West Grove, PA) was diluted in PBS-Tween to a final concentration of 1:40,000, and incubated for 30 minutes (150 μL/well). After 5 washes, wells were incubated for 1 hour with 150 μL ABTS substrate-chromogen solution. The latter was prepared from 50 mg ABTS (2,2′-azinobis(3-ethylbenzothiazoline)-6-sulfonic acid; Boehringer, Mannheim, Germany) dissolved in 100 mL of ABTS-buffer (phosphate-citrate-sodium perborate solution, pH 4.6; Boehringer). The optical density (OD) was read at 415 nm (Multiskan RC version 6.0, Labsystems, Helsinki, Finland). The OD of the antigen-free control well was subtracted from the OD of the corresponding antigen containing well, the average was taken, and the result expressed as the percentage positivity of the positive control serum included in the plate. The sample was considered positive if the percentage positivity was above 30%.

The Immune Trypanolysis (ITL) assay [18] is an antibody-mediated complement lysis test of trypanosomes that can be used to confirm the presence of gambiense specific antibodies. It was performed as described by Camara *et al*. using live *T*. *b*. *gambiense* parasites [13]. In brief, two discs of 6 mm diameter were punched out and incubated overnight in 40μl guinea pig serum. The eluate was mixed with an equal volume of guinea pig serum, to which 50 μL of a 10^7^ trypanosomes/ml suspension prepared from infected mouse blood was added. After incubation at room temperature, the suspension was examined by microscopy (x250). ITL was considered positive when more than 50% of the trypanosomes were lysed. ITL was carried out with both LiTat 1.3 and 1.5, for all ELISA positive samples and a random selection of 10% ELISA negative samples at the *Institut National de Recherche Biomédicale* (INRB) in Kinshasa. For Quality Control purposes, initially all ELISA positive samples and 5% of the ELISA negative samples were further shipped to the Institute of Tropical Medicine (ITM), Antwerp, Belgium. As anomalies were detected (incorrect execution of the procedure and inadequate storage), from 2018 onwards all ELISA positive samples and a random selection of 10% ELISA negative samples were no longer processed at INRB but sent to ITM for ITL analysis.

### Data analysis

Statistical analysis was performed with STATA, version 14 (StataCorp. College Station, USA). Descriptive statistics are presented using frequencies, proportions and the exact (Clopper-Pearson) 95% Confidence Intervals (95%CI). The mean ± standard deviation (SD) was computed for normally distributed data, else the median and interquartile range (IQR) is presented.

Participation rates (i.e. number of inhabitants enrolled in the study among estimated total number of inhabitants) per village were calculated using known total population estimates. In the health district of Kimpese precise population estimates were available for the area covering the population enrolled in a Health and Demographic Surveillance System (HDSS) (Phanzu *et al*., manuscript in preparation). It monitors a demographic cohort of about 60,000 inhabitants. For the remaining areas, population estimates were retrieved from the health district medical manager, however they were less precise and sometimes missing. We constructed an age pyramid of the study population residing in Kongo Central province and compared it to the age pyramid generated by the HDSS, the study population residing in Haut Katanga province were excluded from this analysis because data on the age distribution of the general population were unavailable.

## Results

### Study participation

**Fig 4:**
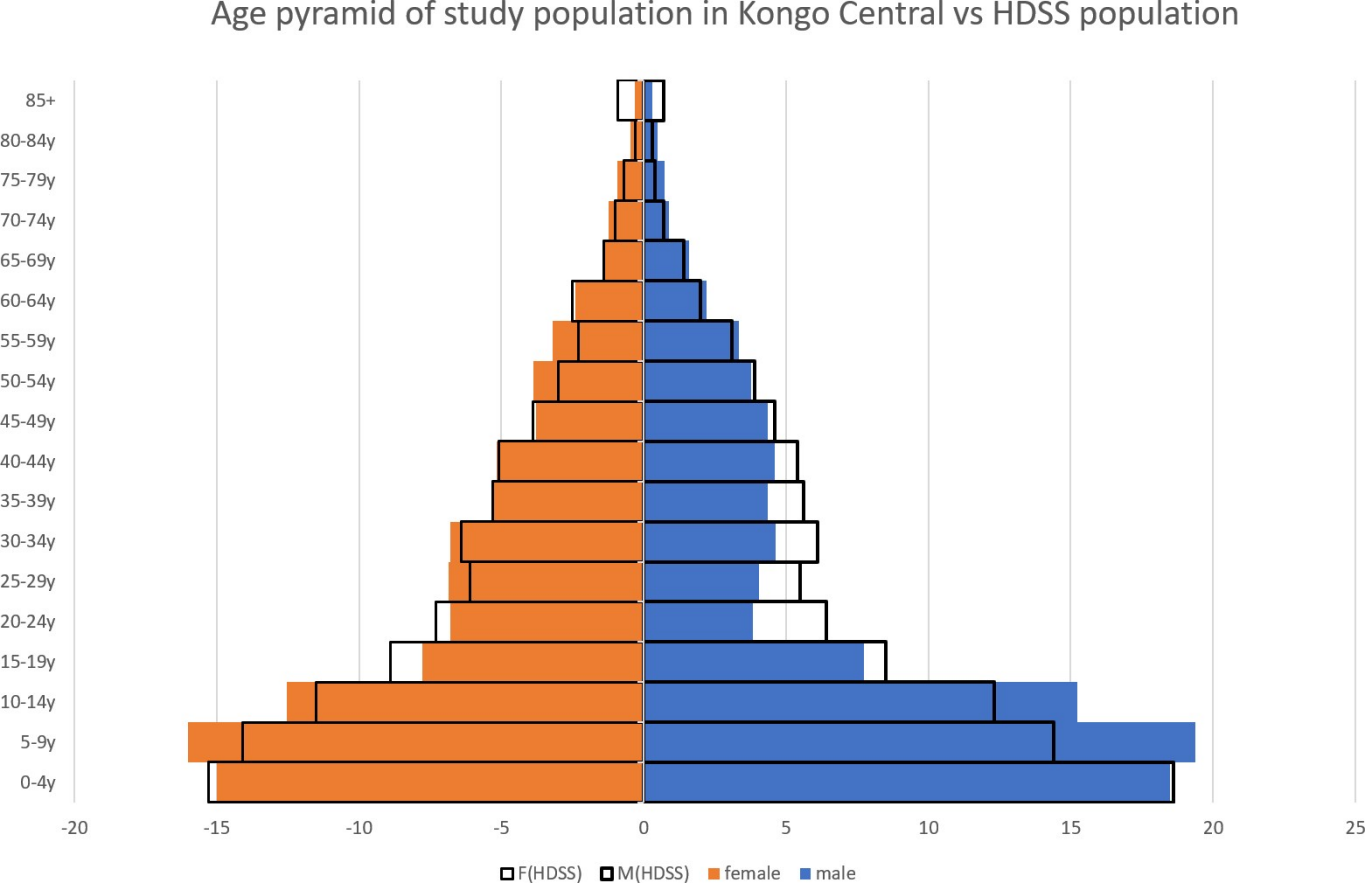
Age distribution of female (n = 6,286) and male (n = 5,360) individuals participating in a sero-surveillance study collecting Dried Blood Samples in Kongo Central province, DRC (2017–2019) compared to the age distribution of the HDSS population in the health district of Kimpese, Kongo Central (2018). HDSS = Health and Demographic Surveillance System.

In total, 11,645 persons were enrolled between November 2017 and August 2019. The proportion of females was 54% and the median age was 15 years old (IQR: 6–35), ranging from one to 98 years old. At village level, the participation rates were variable, but significantly higher in Kongo Central compared to the non-endemic health area in Haut Katanga province. The median participation rate ranged from 63.6% (IQR: 37.8–81.6) in Malanga, 73.0% (IQR: 67.0–86.0) in Lovo, to 24.5% (IQR: 12.7% - 38.7%;) in Adra41. For the other health areas, precise participation rates could not be calculated as village population data were incomplete. The age pyramid of the study population residing in the Kongo Central province was compared to the one from het HDSS population of Kimpese (Fig 4). It shows that coverage of children up to 15 years old, women, elderly of 60 years and older could be considered very high. However, it could be assumed that a considerable part of the young male population was missed, between ages 20 and 45 in particular. Refusal to participate was rare within the HDSS observation area; non-participation was mainly caused by the fact that not all household members were present at the moment the study team passed, although refusal cannot be entirely excluded.

### ELISA and TL testing results

In total 11,535 samples were processed for ELISA out of 11,642 collected (99%), i.e. 46% from male participants and 54% from female participants. In total 97 samples were ELISA positive, 27 from male and 70 from female participants. The median age for ELISA positives was 37 years (IQR: 16–49). In the endemic zone, ELISA positivity was significantly higher 1.34% (95%CI: 1.04–1.64) compared to the previously endemic zone and the control zone, ELISA positivity was 0.34% (95% CI: 0.13–0.55) and 0.37% (95% CI: 0.15–0.60) respectively (Table 2).

**Table 2:** Overview of ELISA/*T*.*b*. *gambiense* and Immune Trypanolysis results in health areas in DRC (2017–2019).

Health Areas	#Samples	ELISA+(n)	ELISA+(%)	95% CI	TL+(n)
Endemic (Kimpese & Nsone Mpangu district)	5,675	76	1.34%	1.04%– 1.64%	2
Previously endemic (Kimpese district)	2,923	10	0.34%	0.13% - 0.55%	0
Control (Kafubu district)	2,937	11	0.37%	0.15% - 0.60%	*
	11,535	97	0.84%	0.67% - 1.01%	2

ELISA+: Elisa positive samples, ITL+: Immune Trypanolysis positive samples

*ITL analysis was not performed.

**Fig 5:**
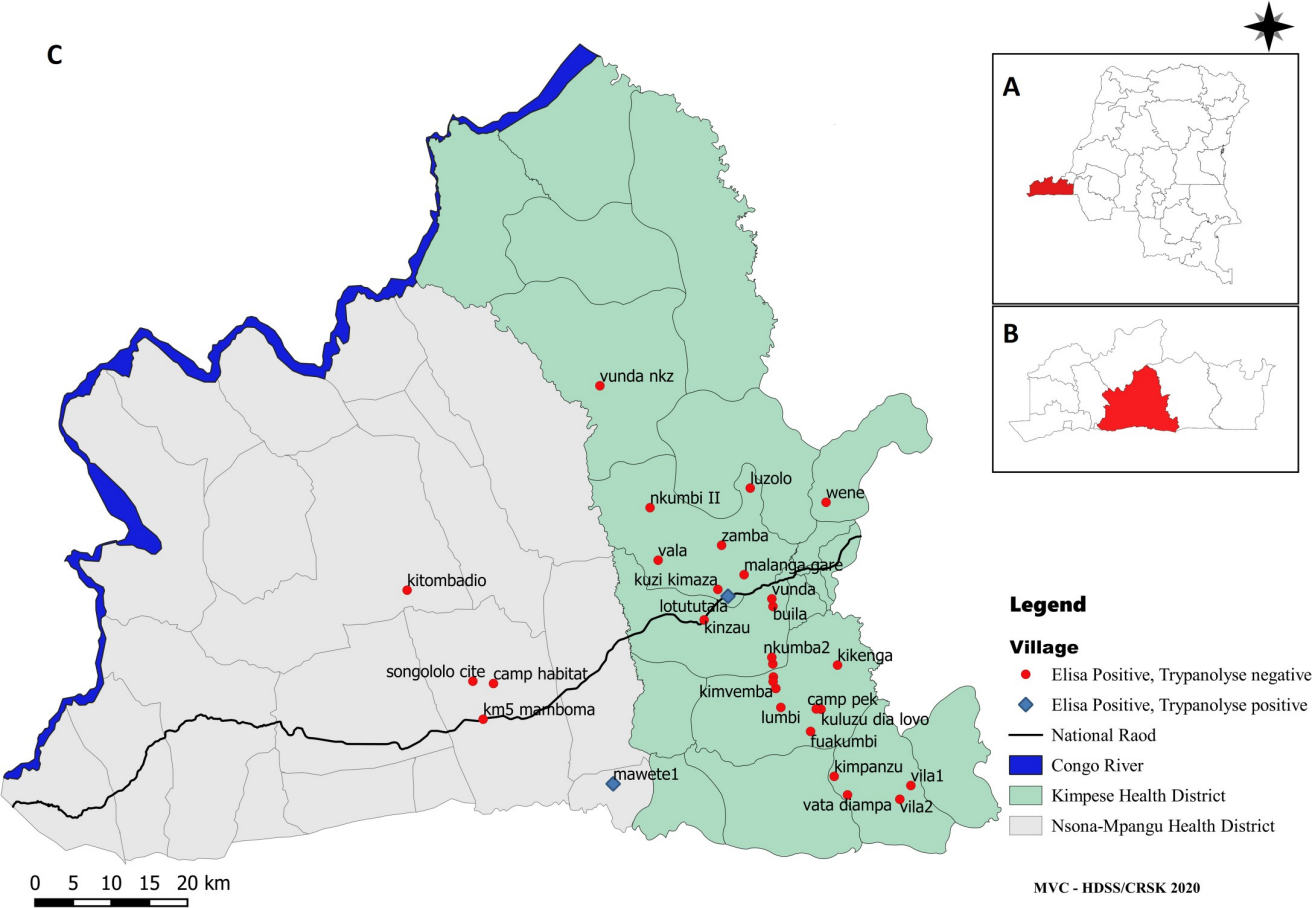
Mapping of the study area in Kongo Central province. **A:** The Democratic Republic of the Congo with Kongo Central province highlighted. **B:** Kongo Central province, the study area highlighted. **C:** Map of Nsona Mpangu and Kimpese district, villages are shown with positive ELISA and/or TL result. Map created using QGIS 3.16 based on https://www.naturalearthdata.com/downloads/10m-cultural-vectors/ and https://www.diva-gis.org/gdata.

In the endemic and previously endemic areas, all 86 (100%) ELISA positive samples and 269 (3%) ELISA negative samples were tested by ITL. This was less than the planned 10% and explained by inadequate storage of the samples, insufficient blood spot left for processing and non-compliance to the ITL standard operating procedure. Among the ELISA positives, only two samples also had a positive ITL result. Both samples came from females >60 years residing in the endemic zones. The first appeared to be a former HAT patient diagnosed and treated in 2008. Unfortunately, the second ITL positive individual had deceased prior to the follow-up visit. All ELISA negative samples that were also assessed by ITL were negative. The map presenting ELISA and ITL positivity per village in Kongo Central province is shown in Fig 5.

In the Kafubu non-edemic district, 11 samples were found ELISA positive but none were processed for ITL, for the reasons mentioned above. All 11 individuals with a positive ELISA result were revisited. Eight of them agreed to undergo a full HAT diagnostic testing, which was negative for all.

## Discussion and conclusion

The results of this study confirm earlier indications that the ELISA/*T*.*b*. *gambiense* technique using dried blood spots is a promising candidate as screening test for a surveillance system in low prevalence settings [12]. In 2019, a total of 0.7% CATT/RDT positives was reported at DRC country level. (PNLTHA: annual report 2019). In the present study, the global seropositivity rate for the ELISA screening was comparable (0.84%). ELISA positivity rates were significantly higher in endemic areas (1.34%) compared to previously endemic (0.34%) and non-endemic areas (0.37%). ITL was used as the second step in the testing algorithm and the two TL-positives that were found in this study were identified in the endemic area. The proportion of assumed false positive ELISA results in the non-endemic area was similar to that of the previously endemic health areas where HAT had not been reported since 2007, thus suggesting a specificity of 99.6%. This result is in line with previously reported specificity of ELISA/*T*.*b*. *gambiense* on DBS ranging from 96.5%-99.8% [12]. The present study also demonstrated that transportation of DBS samples from remote villages for testing by ELISA at regional laboratories is a feasible option.

The ELISA/*T*.*b*. *gambiense* exists since a few decades and has several assets. It has a high throughput format, with >100 tests conducted per laboratory technician/day in the context of this study. High sensitivity (98.7; CI 93.1–100.0) and specificity (99.2; CI 95.7–100.0) have been reported on serum [5], on DBS the sensitivity has been reported to range from 82.8%-96.9% [12]. The cost has been roughly estimated (filter paper, consumables and labor cost included) at 1 € per test, it is more expensive than CATT (0.5€) but cheaper than the RDT Sero-K-Set (1.7 €). A costing study is required to properly evaluate and compare true costs of these tests when applied under the different surveillance strategies. Dried Blood Spots (DBS) have the advantage that they are relatively easy to collect, transport and store. In the current context of DRC (i.e. a weak health system with limited routine laboratory facilities), ELISA is not an appropriate tool for integrated HAT control at the district health system level. However making ELISA platforms available in regional laboratories, could also play a role in disease control beyond HAT, as the ELISA technique is used for the detection of many pathogens.

A major disadvantage of ELISA/*T*.*b*. *gambiense* in HAT control, is that a positive result requires additional testing. In the present study, the overall positivity rate was 0.84% and only two out of 86 (2.4%) ELISA/*T*.*b*. *gambiense* positives assessed, had a positive ITL result. This is in the same order of magnitude as the percentage of confirmed cases (1.8%) among CATT or RDT positive individuals (0.7%) during routine active population screening by mobile teams, reported in 2019 for the DRC (PNLTHA, Annual report 2019). In a set up with remote testing of DBS, preferably the follow-up test should be performed in the same laboratory, using the same sample, to avoid additional sampling and transport.

In the present study, ITL was used as a second step in the testing algorithm. ITL is recognized by the World Health Oganization (WHO) as the reference test to confirm exposure to *T*.*b*. *gambiense* because it combines high sensitivity with high specificity [5]. The same DBS used for ELISA can also be used for ITL, as shown in this study. However ITL has a number of serious drawbacks. It has a low throughput, is expensive (5–7 €) and complex but above all, it is not readily available. Worldwide only three specialized laboratories are capable to perform ITL. This is partly explained by the necessity to maintain clones of trypanosome populations expressing the Variant Antigen Types (VAT) LiTat 1.3 and 1.5 *in vivo* with consequent exposure risks for the handlers [14]. The technique also requires specific training and quality assurance. Even in these centres, errors can occur as illustrated in the present study with the main causes being: non-compliance to the ITL standard operating procedure due to insufficient training and inability to perform ITL due to insufficient blood on the filter paper. The above elements explain why, in the absence of reliable alternatives for ITL, the use of remote testing based on an algorithm of ELISA/*T*.*b*. *gambiense* followed by ITL has so far been limited. Studies mainly focused on the potential of ELISA/*T*.*b*. *gambiense* for post elimination surveillance and looked for thresholds that would indicate re-emergence of gHAT [12]. However a possible alternative for ITL has now been developed. The new *T*.*b*. *gambiense*-inhibition ELISA (g-iELISA)[19], appears to have similar sensitivity and specificity as ITL but with additional assets. g-iELISA is based on the principle that antibodies in the blood of gHAT patients inhibit the binding of monoclonal antibodies to VAT-specific epitopes on the VSGs of *T*.*b*. *gambiense* variants LiTat 1.3 and LiTat 1.5. The test is already commercially available at a relatively low cost (3€/test)[19]. The equipment needed is the same as for other ELISA assays, the same DBS sample can be used and staff already trained for ELISA would require limited additional training. As demonstrated in the present study, the use of ELISA in regional laboratories is feasible. The availability of the g-iELISA opens new perspectives for the gHAT surveillance. However, further investigation is needed to establish its sensitivity, specificity and cost-effectiveness in field conditions.

With the arrival of new oral drugs, effective on both stages of disease [20], the pressure is rising to move from a *screen-confirm&treat* towards a *screen&treat* strategy. The method described in this study offers future perspectives when the candidate compound acoziborole, that may cure both stages of HAT with a single oral dose, becomes available. For the moment ITL cannot be used to guide a treatment decision as parasitological confirmation is still required. The arrival of an effective and safe single dose oral drug might allow treatment based on a serological test result, thus becoming a game-changer. Sample collection and treatment would still require visiting villages but the size of teams could be drastically reduced. Small motorcycle based teams could be used for a variety of tasks, planning can more flexible, and they may also reach more remote areas that are difficult to access for traditional mobile teams moving around by car and with a lot of equipment. This approach still requires that field teams return to the village for further examination, and if necessary provide medical support and/or treatment, to those found to be positive on ITL (or on g-iELISA in the future). A strategy to easily retrieve these individuals is therefore necessary, for example gathering contact information or capturing geo-localisation points. Our study showed that this is feasible and only a minority of villages (2 out of 67) needed a follow-up visit.

Centralization of testing and the use of DBS would also facilitate quality assurance, which has posed major challenges for g-HAT diagnosis in the past [21]. The results of the routinely used serological screening tests and parasitological confirmation techniques cannot be stored, implying that, at best, quality assurance was based on internal quality assurance by another member of the team. With staff afraid of losing their job, the risk of false positive reports is increasing, as observed in the northern part of DRC [22]. Therefore, in recent years a digital quality assurance system has been introduced in DRC through a tablet or smart phone application that allows taking pictures of serological test results and recording videos of living trypanosomes in microscopy confirmation tests. The data are uploaded to a central database on a network associated server and checked at a centralised point. This system proved feasible but adds to complexity, requiring additional equipment and internet connection [21]. In contrast, quality assurance of ELISA in a centralised laboratory is relatively easy to organise both on the spot or in another laboratory.

A crucial success factor for such an approach is appropriate sample collection, storage and transport of DBS. Theoretically, DBS can be collected by staff with limited training and equipment, and can be dried, stored and shipped at ambient temperatures. To warrant minimal degradation of antibodies on the DBS, they must be kept dry by timely replacing silica gel, in which case they can in principle be kept for several months. However, for prolonged storage, longer than 28 days, the systematic review by Amini *et al*. indicated that storage at -20°C is the optimal solution [23]. In g-iELISA, work is ongoing to overcome stability problems (short shelve life of 8 months on 2–8°C) with an impact on storage conditions and on looking for alternatives for filter paper to collect DBS as the number of discs required for testing is high [19].

Selecting the right populations has always been a major challenge in HAT screening, with children and elderly overrepresented in comparison with young adults [24]. Possible explanations are that the latter population group is not willing to waste time and/or have their daily activities outside the village. On the other hand, population data are based on estimates that are not always reliable. Collecting DBS should be organized in a way as to maximize inclusion of high-risk groups. This is feasible because the procedures for sample collection are logistically lighter than those of the traditional mobile screening teams. From the point of view of the people being screened, the procedure still requires a finger prick but waiting times can be reduced to a minimum when teams pass door to door with the possibility of working with appointments.

Nevertheless, our data on participation compared to population data of a nearby HDSS, suggest that voluntary participation of young male adults was still lower than for other groups. The participation rate was also very low in the Adra41 Health Zone. The population was very reluctant to participate because they feared of being tested for other diseases such as HIV. This area never reported HAT cases up to now, therefore the disease is quite unknown and not among the health concerns of the population. As the disease becomes rarer, even in currently endemic areas, it will become more challenging to maintain an interest for the disease in the at risk population.

The limitations of the study are mainly related to the tests performances. In absence of a gold standard in combination with low HAT prevalence, it is difficult to assess the actual sensitivity and specificity of the tests. ITL was considered the confirmation test with 100% specificity and a sensitivity close to 100% [13], therefore there is still a small probability that ITL failed to detect antibodies in some. Another crucial factor that can influence the test result are the storage conditions. Filter papers should be kept dry by timely replacing silica gel when is becomes humid to avoid antibody degradation. This requires dedicated lab-technicians and constant monitoring of the samples. Even though procedures are intended to limit errors, it cannot always be warranted at 100% as this study also suffered from losses due to inadequate storage conditions among other things. As a result we were unable to confirm by ITL that in the non-endemic area the ELISA positives where all truly false positives. However the results indicate that in the endemic area the ELISA followed by ITL performed as well as the traditional combination CATT/RDT–mAECT. ITL-positives, thus potential HAT cases, were only found in the endemic zones providing strong indications that the method can detect active transmission in a given area.

After decades of applying a HAT control strategy, based primarily on large mobile teams screening endemic villages, over the last 5 years new modalities have been piloted in the HAT control strategy of the DRC, including different types of mobile teams [10] and rapidly expanding vector control [25]. However, in order to reach the interruption of g-HAT transmission by 2030, additional innovation is required for active and passive case detection. For passive case detection, integration of HAT diagnostic activities into the national health system is a strategy that is currently proposed, while for active screening, innovation in surveillance is carefully considered [3]. The present study showed that screening based on DBS collected on filter paper and testing in regional laboratories might be considered as an alternative strategy in the future. This could become a feasible option in HAT endemic areas, traditionally visited by mobile teams. It has also potential for post-elimination surveillance to monitor resurgence and for exploratory surveillance in historic foci that have not been under surveillance in recent years.

## Supporting information

S1 Dataset Supporting data. (DTA)

S1 Label list List of labels. (XLSX)
